# Efficacy of multiple Biomarkers: NGAL, KIM1, Cystatin C and IL18 in predicting pregnancy related acute kidney injury

**DOI:** 10.12669/pjms.39.1.6930

**Published:** 2023

**Authors:** Rubina Naqvi, Nazli Hossain, Sofia Butt, Zeenat Bhellar, Erum Fatima, Sanam Imtiaz, Palwasha Ghulam Moosa, Khawar Abbas, Salma Batool Jafri, Sadia Khan

**Affiliations:** 1 Rubina Naqvi, Dept. of Nephrology, Sindh Institute of Urology and Transplantation (SIUT), Karachi. 74200. Pakistan; 2 Nazli Hossain, Dept. of Obstetrics and Gynecology, Unit II, Ruth Pfau KM Civil Hospital, Karachi. 74200. Pakistan; 3 Sofia Butt, Dept. of Obstetrics and Gynecology, Unit II, Ruth Pfau KM Civil Hospital, Karachi. 74200. Pakistan; 4 Zeenat Bhellar, Dept. of Obstetrics and Gynecology, Unit II, Ruth Pfau KM Civil Hospital, Karachi. 74200. Pakistan; 5 Erum Fatima, Dept. of Obstetrics and Gynecology, Unit II, Ruth Pfau KM Civil Hospital, Karachi. 74200. Pakistan; 6 Sanam Imtiaz, Dept. of Nephrology, Sindh Institute of Urology and Transplantation (SIUT), Karachi. 74200. Pakistan; 7 Palwasha Ghulam Moosa, Dept. of Nephrology, Sindh Institute of Urology and Transplantation (SIUT), Karachi. 74200. Pakistan; 8 Khawar Abbas, Dept. of Lab Sciences, Sindh Institute of Urology and Transplantation (SIUT), Karachi. 74200. Pakistan; 9 Salma Batool Jafri, Dept. of Lab Sciences, Sindh Institute of Urology and Transplantation (SIUT), Karachi. 74200. Pakistan; 10 Sadia Khan, Dept. of Lab Sciences, Sindh Institute of Urology and Transplantation (SIUT), Karachi. 74200. Pakistan

**Keywords:** Acute Kidney injury (AKI), NGAL, KIM-1, Cystatin C, IL-18

## Abstract

**Objective:**

Several biomarkers like NGAL, KIM-1, IL-18, and Cystatin C has been previously reported as reliable marker to predict AKI. However, their predictive accuracy varies widely. We aim to observe the efficacy of multiple markers, NGAL, KIM-1, Cystatin C and IL-18, in obstetric population who are at risk of developing AKI.

**Methods:**

This prospective study was carried out between June 2021 to March 2022 at Department of Obstetrics & Gynecology Unit II, Ruth Pfau KM Civil Hospital and Sindh Institute of Urology & Transplant (SIUT), Karachi Pakistan. On women brought to OBGYN-ER with the diagnosis of hemorrhage (antepartum and postpartum), hypertension (pre-eclampsia and eclampsia) and sepsis. The urine samples and 3cc blood was collected at the time of admission, blood sample processed for biochemistry at time of admission and repeat blood samples for serum creatinine at 24 and 48 hours. Urine was stored at -80ºC and later evaluated for NGAL, KIM-1, Cystatin C and IL-18. Serum Cystatin C was also processed for the time zero sample. The biomarkers were tested using ELISA assays.

**Results:**

A total of 149 women were included in the study, 83% of these women were non-booked. Twenty-six (17%) women developed AKI. Serum Cystatin C, urinary Cystatin C and urinary NGAL were found significantly raised in women who developed AKI. While KIM-1 and IL-18 were not raised to statistical significance in this population. However, urinary KIM-1 along with urinary Cystatin C were significantly raised in women with positive quick sequential organ failure assessment (qSOFA).

**Conclusion:**

This study validates the use of serum and urinary Cystatin C and urinary NGAL as highly predictable biomarkers for the development of AKI and nullifies urinary IL-18 and KIM-1 in this regard.

## INTRODUCTION

Pregnancy- associated conditions like preeclampsia, eclampsia, hemorrhage and sepsis are associated with acute kidney injury (AKI).^1^,^2^ Women who develop AKI have a worse clinical outcome, for example, prolonged hospitalization, temporary need for renal replacement therapy or in some cases end-stage renal failure.^1^ Current guideline of AKI are based on serum creatinine level and decline in urine output and are standard markers for diagnosing AKI.^3^

Unfortunately, in AKI rise in creatinine is delayed by the timing of injury and changes in glomerular filtration rate (GFR). There has been a search for biomarkers that can predict AKI early to intimate timely intervention. Neutrophil Gelatinase Associated Lipocalin (NGAL) is a small protein released from the granules of neutrophils. Its presence in tissue is identified in the breast, colon, and kidney and is increased in epithelial injury and in neoplastic conditions. Serum and urinary levels are found to be significantly raised in conditions affecting the kidney. The rise in urinary NGAL levels is reported to be directly related to the severity of AKI.^4^ The rise in NGAL is seen before the rise in serum creatinine levels, and hence is considered a marker of acute ischemic injury to the kidney has been identified in emergency room settings, after cardiac surgery and after chemotherapy.^4^,^5^ In a case-control study comparing healthy women with pre-eclampsia women, urinary NGAL, showed a significant rise in women with eclampsia, but not with pre-eclampsia (PET).^6^

In another cohort of more than 50 women admitted to the intensive care unit, serum NGAL was found raised in eclampsia, mortality or increased stay in intensive care.^7^ In another case-control study, with age, gestational age matched controls, serum NGAL was found raised in women with pre-eclampsia, compared to normotensive women.^8^ This rise in NGAL has been considered to be associated with inflammation, ischemia and changes due to oxidative stress induced by hypertension during pregnancy.

Other biomarker that is found to be increased in renal injury is Kidney Injury Molecule1 (KIM-1). It is excreted in minor amount during normal conditions as well, but its excretion is reported to be markedly raised in ischemic renal injuries.^9^,^10^ IL-18 (previously known as gamma interferon inducing factor), is a pro-inflammatory cytokine, that has the ability to promote the development of T helper one (Th1) cells and is expressed by activated macrophages and dendritic cells. Elevated urinary IL-18 has been reported in humans in acute tubular necrosis.^11^ Significantly higher levels of serum IL-18 have been reported in women with preeclampsia, whether it was mild or severe.^12^

Cystatin C; a protease inhibitor, is freely filtered by glomerulus and reabsorbed but not secreted by the renal tubules. Urinary cystatin C levels have been reported elevated in tubular dysfunction.^13^ On view of the previous findings we have analyzed multiple markers NGAL, KIM-1, Cystatin C and IL-18 to predict AKI in selected women at time of child birth, who have risks like hemorrhage, sepsis, pre-eclampsia or eclampsia; along with standard markers i.e., rise in creatinine or decline in urinary output.

## METHODS

This prospective study was conducted at the Department of Obstetrics & Gynecology Unit II, Ruth Pfau KM Civil Hospital and Sindh Institute of Urology & Transplant (SIUT), Karachi Pakistan. The study period was from June 2021 to March 2022. Women admitted in the ER of the above unit with the diagnosis of hemorrhage (antepartum and postpartum), hypertension (pre-eclampsia and eclampsia) and sepsis were included in the study. The diagnosis of hemorrhagic shock, both antepartum and postpartum was made by calculating Shock Index (SI), which is calculated by dividing heart rate to systolic blood pressure. Pre-eclampsia was defined as systolic blood pressure of > 140 mm Hg and diastolic blood pressure of > 90 mm Hg, along with proteinuria. Eclampsia was defined as the presence of tonic-clonic convulsions. Sepsis was identified with a positive modified or quick sequential organ failure assessment (qSOFA) score.

Quick Sofa Score (q SOFA): includes hypotension (systolic blood pressure ≤100mm Hg, respiratory rate of ≥ 22 /min, and altered mental status, (which was assessed by Glasgow Coma Scale). This scoring system was introduced in 2016, as the new definition of Sepsis. The score varies from 0-3, a score of two is considered as positive qSOFA. The specificity of qSOFA, outside the intensive care setting has been found to be around 84%.^14^

AKI was defined according to kidney disease improving global outcome (KDIGO) guidelines.^3^ Exclusion criteria included patients with established diabetes mellitus and chronic hypertension or any other systemic disease or co-morbid, which can give rise renal dysfunction. The sample size was calculated using standard methods of estimation described in literature.^15^ In order to detect area under the curve of receiver operating characteristic curve (AUC-ROCC) with power of 80% and 95% confidence level, a total of 97 patients were required for the study.

The study was approved by the Institutional Review Board of Dow University of Health Sciences (DUHS) and the Ethical Review Committee of SIUT (Ref: IRB-2104/DUHS/Approved/2021/651, Dated: December 30, 2021). Co-investigators approached patients, in the delivery suite and if they fulfilled the inclusion criteria, were asked for informed consent. The demographic details, clinical profile and maternal and perinatal outcomes were recorded on a predesigned proforma. The Study population was selected under strict definition criteria given above thus there was no chances of selection biases in the population. Once the eligibility criteria were fulfilled, 50 cc urine samples were collected at time of admission, and 3cc blood was collected at admission and processed for CBC and biochemistry. Repeat 3cc blood samples were taken at 24 and 48 hours to analyze serum creatinine only. Urine output volume was measured on day zero next day and day after (till 48 hours).

The urine samples from time zero were centrifuged and stored at -80ºC, for further analysis. Blood sample from time zero was analyzed for CBC, urea, creatinine, liver functions, uric acid, LDH and Cystatin C; whereas urinary sample was evaluated for NGAL, KIM-1, Cystatin C and IL-18. The biomarkers were assayed using ELISA technique. For Cystatin C TRIMERO Diagnostics kit for IMMAGe 800 was used, which included Controls Accuracy 360. For NGAL Abbots ARCHITECT based kits were used. For KIM-1 and IL-18 BT Lab (Bioassay Technology Laboratory) kits were used which have ELISA based assays. All the tests were run in duplicate.

### Statistical analysis:

The variables as mean ± standard deviation assessed with t-test for comparison for normally distributed and as median with 25^th^ to 75^th^ percentiles with Mann-Whitney U test for skewed data. The ability of markers to detect AKI was assessed by non-parametric calculation of the area under the curve for receiver operating characteristic curve (AUC-ROCC). A two-sided *p-value* of ≤ 0.05 was considered statistically significant. Prism version 9.3.1for Mac OS (www.graphpad.com), was used for all analysis except for logistic regression. The logistic regression and correlation matrix analysis were done on STATA version 16.1 for Mac OS (www.stata.com).

## RESULTS

Between June 2021 to March 2022, a total of 149 women at this OBGYN unit, who fulfilled the criteria of selection, that is, were at risk of developing AKI, were included in the study. The demographic details and baseline clinical and laboratory data in the study population is shown in Table-I. The majority, that is 123 (83%) of the women did not receive any form of antenatal care from health care providers. Of the 149 women, 13(9%) had pre-eclampsia, 27(18%) had eclampsia, 72 (48%) had antepartum hemorrhage (APH) and another 10 (7%) had post-partum hemorrhage (PPH), positive qSOFA scores were detected in 66(44%) women.

**Table-I:** Demographic characteristics and baseline laboratory values (n=149).

Parameter	mean± sd	Median (IQR)	range
Age of patient (year)	27.21± 5.33	27 (20.5-32)	18-40
Gestational age (weeks)	32.27±7.21	34 (29.5-37)	3-40
Systolic BP (mm Hg)	126.48±32.18	130 (100-150)	60-200
Diastolic BP (mm Hg)	81.72±21.66	80 (62.5-100)	30-140
Hemoglobin (gm/dl) (11.5-15.4G/dl)	8.81±2.26	9 (6.75-9.85)	3.8-19.2
TLC (10^9^/L) (4-11 x103/μl)	17.57±6.85	16.32 (12.87-25.13)	2.90-46.56
Platelet (10^9^/L) (150-400 x103/μl)	179.69±88.55	171(55.5-191)	24-456
Urea (mg/dl) (15-39mg/dl)	26.23±16.60	23 (37-61)	8-149
Creatinine time zero (mg/dl) (0.5-1.5)	0.75±0.47	0.61(1.32-2.04)	0.18-2.87
Creatinine 24 hours (mg/dl) ) (0.5-1.5)	0.87±0.89	0.54 (1.13-2.72)	0.21-5.33
Creatinine 48 hours (mg/dl) ) (0.5-1.5)	0.93±1.20	0.56 (0.9-4.43)	0.41-6.35
S. Bilirubin total (mg/dl) (0.2-1.0 mg/dl)	0.63±0.55	0.47 (0.18-0.5)	0.12-3.84
AST (U/L) (10-42 U/L)	80.83±213.89	40 (43-142)	8-2193
ALT (U/L) (10-40 U/L)	45.50±177.81	17 (17-52)	2-1833
LDH (U/L) (<247 U/L)	567.44±638.02	383 (331.5-1301)	113-4542
Uric Acid (mg/dl) (3.5-7.0 mg/dl)	6.18±2.64	5.91 (5.23-11.25)	1.97-15.73

TLC= total leucocyte count, AST=aspartate transaminase, ALT=alanine aminotransferase, LDH= lactate dehydrogenase.

There were 26 (17.44%) women who developed AKI at 24 hours according to KDIGO definition. Different parameters compared between those who developed AKI and those who did not is given in Table-II. Statistical values of the studied biomarkers in AKI and non-AKI groups are given (Table-III).

**Table-II:** Characteristic Comparison in patients AKI vs no AKI.

Characteristics	AKI (n=26) mean± sd	No AKI (n=123) mean± sd	P-value
Age of patient (year)	28.53± 6.738	27.01± 4.90	0.386
Gestational age (weeks)	33.03v 5.56	32.09± 7.46	0.925
Number of pregnancies	1-9	1-10	0.306
Booked	19 %	16.9%	0.739
Shock index	0.831± 0.313	0.869 ±0.317	0.434
Hemoglobin (gm/dl)	7.75± 1.95	9.02± 2.22	0.004
TLC (10^9^/L)	20.85± 9.14	16.87± 5.93	0.027
Platelet (10^9^/L)	129.96± 90.88	189.59± 83.30	< 0.001
AST (U/L)	216.15± 464.68	51.75± 63.40	< 0.001
ALT (U/L)	140.34± 397.38	25.11 ±43.21	0.041
LDH (U/L)	1153.84± 1161.02	441.42± 334.29	< 0.001
Uric Acid (mg/dl)	7.68± 3.24	5.86± 2.35	0.010
Positive qSOFA	12 (45.5%)	67 (54.5%)	0.019
Ante-Partum Hemorrhage	14 (53%)	58 (47%)	0.533
Post-Partum Hemorrhage	16 (60%)	49 (40%)	0.055
Serum creatinine (mg/dl) at 24 hours	2.085±1.104	0.714±0.744	<0.0001
Serum creatinine (mg/dl) at 48 hours	2.679±1.849	0.752±0.991	<0.001
Urine output at 24 hours	0-310 ml/24 hours	1200-4000 ml/24 hours	<0.0001
Urine output at 48 hours	0-70 ml/24 hours	1600-4000 ml/24 hours	<0.0001

**Table-III:** Statistical significance of different biomarkers (p values).

Biomarker (Reference range)	AKI (n=26) mean± SD (range)	No AKI (n=123) mean± SD (range)	P value
Serum Cystatin C (0.47-1.03 mg/l)	2.46±1.03 (1.24-5.30)	1.26±0.52 (0.30-3.63)	<0.0001
Urinary Cystatin C (<1mg/l)	8.66±24.33 (0.16-99.00)	2.20±4.74 (0.06-27)	<0.001
Urinary NGAL (<131.7 ng/ml)	1264.33±1941.56 (6.10-6000)	118.83±238.49 (0.00-1324)	<0.0001
KIM-1 (up to 0.5ng/ml)	1.28±0.42 (0.399±2.258)	1.35±0.58 (0.39-3.94)	0.453
IL-18 (0.01-0.14ng/ml)	19.38±9.55 (9-46)	20.56±12.14 (1-64)	0.692

**Fig.1:**
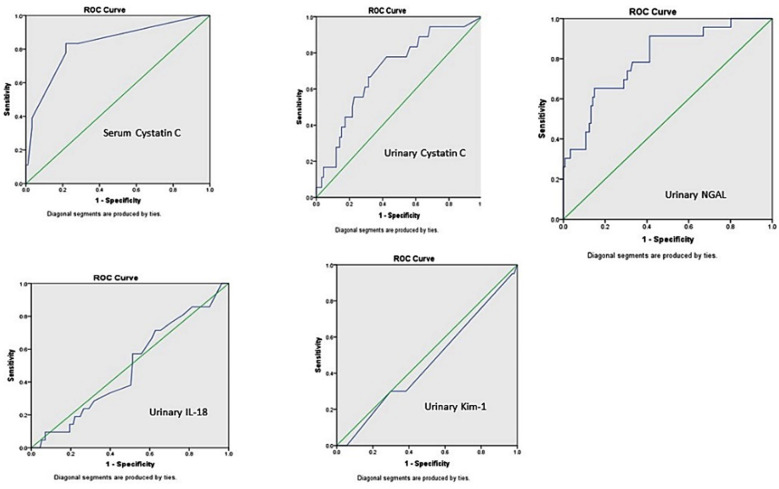
AUC-ROCC showing probability of different biomarkers with development of AKI.

The Mann Whitney U test was applied and drawing ROC curves were done. The urinary NGAL levels found significantly raised (p = <0.0001) in women who developed AKI with an AUC-ROCC value of 0.798. Serum cystatin C also shown high significance (p = <0.0001) and its AUC-ROCC value was 0.898. Urinary cystatin C was also a significant predictor for AKI, with p value of 0.001 and AUC-ROCC of 0.702. Whereas, urinary IL-18 showed p value 0.692 and AUC-ROCC of 0.512 and KIM-1 p value of 0.453 and AUC-ROCC of 0.504 (Fig.1). In order to construct AUC-ROC curves, sensitivity and specificity were calculated for different scores for serum Cystatin C, urinary Cystatin C and urinary NGAL.

**Fig.2:**
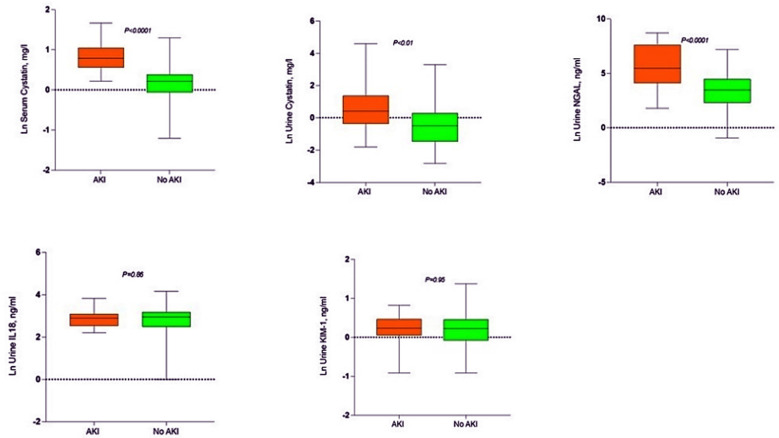
AKI vs no AKI. Box plot showing Natural Log (Ln) transformed copies at zero time (whiskers represent min/max value).

The sensitivity, specificity, positive predictive value (PPV) and negative predictive values are shown in (Table-IV) for these markers. The traditional range reported in the literature for serum cystatin C is 0.47-1.03 mg/l, in our study at cut-off of 1.2 specificity was 96% and PPV was 83%. For urinary cystatin C; reported in literature as <1mg/l, we found at cut-off of 0.39, the specificity of 92% and PPV of 83%. Urinary NGAL reported as <131.7 ng/ml, we at cut-off value of 40.5 found specificity of 94% and PPV of 78%. As AUC-ROCC for KIM-1 and IL-18 were insignificant their sensitivity and specificity at different cut-off values was not calculated. Natural Log (Ln) transformed copies depicted in box plot for development of AKI vs no AKI (Fig.2). Serum cystatin C and urinary NGAL showed *p-value* of <0.001. Urinary cystatin C was also significant with p value of <0.01, while KIM-1 and IL-18 were insignificant with p value of 0.95 and 0.86 respectively.

**Table-IV:** Diagnostic performance of different biomarkers at selected cut-off scores.

Biomarker	Cut-off score	Sensitivity %	Specificity %	PPV %	NPV %
Serum Cystatin C	1.2	36.58	95.65	83.33	71.73
	2.4	70	89	38.80	96.73
Urinary Cystatin C	0.39	21.10	92.30	83.30	39.10
	1.51	31	88.90	50	78.26
	2.1	30.40	87.35	38.90	82.60
Urinary NGAL	40.5	28.10	93.70	78.26	61.9
	143.1	42.85	92.67	65.21	83.47
	176.3	45.45	92.70	65.20	85.10

**Fig. 3:**
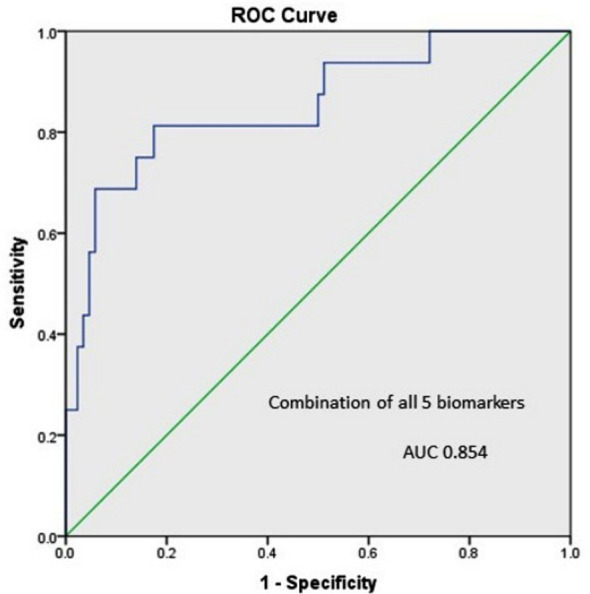
Combination of all five biomarkers to predict AKI.

We also assessed value of combining all five biomarkers in our studied population and found AUC-ROCC of 0.88 (Fig.3), which is slightly lower than serum cystatin C alone (0.89). In logistic regression analysis, serum cystatin C and urinary NGAL remained the significant predictors for developing AKI at 24 hours. Association of markers controlling for PET, sepsis, qSOFA and APH also assessed through this analysis. Serum cystatin C and urinary NGAL were significantly raised in women with pre-eclampsia, while only urinary NGAL in women with ante-partum hemorrhage and eclampsia. Urinary KIM-1 and cystatin C were significantly raised in women with positive qSOFA.

## DISCUSSION

The current study presents use of NGAL, KIM-1, Cystatin C and IL-18 for predicting AKI in population with risk of development of AKI, via the ROC analysis approach. Development of AKI was confirmed by KDIGO criteria of the definition of AKI.^3^ This study was designed in view of our past experience of dealing with a large number of reproductive age women developing AKI after complicated obstetrics^1^,^2^ and thus those who were coming to OBGYN unit and had some risks of developing AKI were considered for inclusion. There was no similar study in literature was found addressing usefulness of set of urinary biomarkers in this particular population, though plasma NGAL^7^,^8^ and both serum and urine NGAL^6^ in setting of pre-eclampsia was studied earlier.

Moyake et al. did not find any significant correlation of NGAL in predicting AKI in women with pre-eclampsia,^6^ the baseline ROC analysis in this particular study could not discriminate between pre-eclampsia patients with or without AKI. Our study revealed NGAL as significantly superior marker for predicting AKI but has not shown significance in selected pre-eclampsia cases. However, urinary NGAL show significant rise in women who had eclampsia or in women with ante-partum hemorrhage. This association of NGAL with pre-eclampsia and eclampsia is similar to earlier reported findings by Moyake.^6^

In past, KIM-1 along with two other markers was studied in HIV associated pre-eclampsia women and was found significantly raised in this population when compared with normal pregnancies.^16^ A meta-analysis has revealed significant superiority of KIM-1 in early detection of AKI, well before rise in serum creatinine, with summary receiver operating curves (sROC) showing an area under the curve of 0.86.^10^ This meta-analysis also highlights heterogeneity in KIM-1 levels with age, population setting and time of measurement of KIM-1. The concluding observation was that KIM-1 has better diagnostic accuracy in infants and children than in adults.^10^ In addition, assays performing KIM-1 analysis can also explain variability in findings and need of more accurate standardization should be considered. In present study we used ELISA assays supplied by Bioassay Technology Laboratory and found significantly higher urinary KIM-1 levels in patients who had abnormal qSOFA score, while in women developing AKI there was no significant rise in our studied population. The qSOFA score has been reported previously as good predictor marker for mortality and multi-organ failure in septic patients.^14^

A study done for contrast induced AKI looking KIM-1 as predictor, has also reported no statistical significance of this biomarker. In this particular study serum creatinine remained more sensitive and specific in patients developing AKI after contrast induced injury, though levels of KIM-1 were high in pre-contrast and post contrast samples.^17^ A meta-analysis performed for role of IL-18 in prediction of AKI via sROC analysis has revealed a pooled diagnostic OR of 5.11 and estimated area under the curve of mean ROC was 0.77. This can be interpreted as application of this marker in diagnosis of AKI should be limited to a certain range.^18^ Our results revealed no statistical significance of this biomarker in prediction of AKI as well as association with any of studied risk factors in our population. Our findings were more in similarity to use of urinary IL-18 reported in adults after cardiac surgery in a study from Australia.^19^

The probability of the combination of KIM-1 and IL-18 in the prediction of AKI has also been reported by Ren et al, where they have shown the significance of these markers individually as well as in combination. The AUC for combination in this study was 0.904.^20^ We did analyzed combination of all our studied markers and AUC was 0.88 in our population, which is slightly lower than serum cystatin C alone (0.89) as predictor for AKI. This could be explained on basis of the fact that two of our five studied biomarkers have remained insignificant in predicting AKI. Royakker et al., in a multi-center study evaluated role of serum and urinary cystatin C in predicting AKI in a heterogeneous intensive care unit population of patients and they reported both serum and urinary cystatin C rise with increasing class of RIFLE. However, predictive ability of these markers in this particular study was poor.^21^

Whereas, a systemic review and meta-analysis done for cystatin C both in serum and urine has clearly shown superiority of serum cystatin C in prediction of AKI in clinical practice. While same meta-analysis reported that urinary cystatin C performed less well in prediction of AKI.^22^ Present study reveals that although both serum and urinary cystatin predict AKI, urinary cystatin performed less well. This finding might be explained on basis of fact that filtered cystatin C completely absorbed through proximal convoluted tubules and will only appear in urine when tubular damage occurs. However, our studied population with abnormal qSOFA scores showed significantly high urinary cystatin C levels.

### Limitations:

Our study was designed to collect urine and blood samples at time of entry at OBGYN unit and repeat blood samples at 24 and 48 hours to check serum creatinine only. Meanwhile their urine output was also measured at these time points that is, 24 and 48 hours. After 48 hours’ women were discharged from hospital and were not followed later. Thus, we are not aware if some of them might have developed AKI in delayed part of time. However, it was not our objective for present study. We only aimed to see levels of biomarkers at time zero and correlate it with development of AKI at 24 or 48 hours. In our studied population only 26 women developed AKI and this was a small number to validate sensitivity and specificity of these biomarkers.

## CONCLUSIONS

In summary, our studied population was different from other published reports in literature as we looked for pregnant women who were at risk of developing AKI, and then we assessed biomarkers at time zero and observed actual development of AKI with established definition of AKI. Our data indicates that urinary NGAL and serum cystatin C individually can predict AKI and represent a novel biomarker for AKI and may enable clinicians to initiate timely intervention in management of AKI and may prevent long term poor prognostic situations in this particular group of patients. We were unable to validate use of KIM-1 at very early stage in development of ischemic AKI, but it can significantly indicate sepsis (which we have assessed through qSOFA scoring). IL-18 though a pro-inflammatory marker remained subtle in predicting AKI or association with other clinical parameters in our study. In future larger study population is required to validate sensitivity and specificity of these biomarkers in this particular population.
